# What’s in a Name?

**Published:** 1893-10

**Authors:** 


### EDITORIAL DEPARTMENT.

F. E. DANIEL, M. D., Editor.
S. E. HUDSON, M. D., Managing Editor.
A. J. SMITH. M. D., Galveston, Associate Editor.

EDITORIAL STAFF:

PROF. J. E. THOMPSON, M. D., Texas Medical College, Galveston; Surgery.
PROF. WM. KEILLER, M. D., Texas Medical College, Galveston; Obstetrics and Gynecology.
PROF. DAVID CERNA, M. D., Texas Medical College, Galveston; Therapeutics.
PROF. A. J. SMITH, M. D., Texas Medical College, Galveston; Medicine.
DR. ISADORE DYER, Tulane University; Dermatology.
DR. R. H. L. BIBB, Saltillo, Mexico; Foreign Correspondent.
DR. ROBT. MORRIS, Charity Hospital, N. O.; Clinical Reports

The JOURNAL is the official organ of the Austin District Medical Society, the West Texas District Medical Society and the Galveston County Medical Society.

### WHAT'S IN A NAME?

"If it is honorable to prescribe according to the law of similars, it is equally honorable to announce that one so prescribes."

The sentiment above quoted is that employed by the editor of the *Medical Century*, in commenting upon the position taken by Professor Obetz, Dean of the Homeopathic Department of the Medical School of the University of Michigan. The *Medical Century* is a homeopathic medical journal. Dr. Obetz's position is that "only those who desire to trade upon a name" demand that schools and organizations be maintained separate for medical sects; and in accordance with such a view he proposes that the two medical schools of the University of Michigan be reorganized so as to form but a single department.

It is no matter of surprise that for the expression of such sentiments Professor Obetz should have had heaped upon his head the maledictions of rabid homeopathists; but it is even less a surprise that he should have offered to him the most kindly expressions of a large number of the most advanced members of the same group of the medical profession. A medical journal of homeopathic affiliation, but of the broader type of journalism and of scientific tone, the *New York Medical Times*, does not hesitate to say that "his proposition will, before very long, be acceptable and accomplished, but we fear not just yet." A correspondent in the same journal acknowledges that the lapses from "pure" homeopathy have come to such a pass that the movement initiated by Prof. Obetz becomes not only explicable, but "as necessary and inevitable as the results of gravitation in the physical world." Nor should there, when such a spirit of conciliation is exhibited, be denied by the regular profession an opportunity for its fuller development. In the nature of its constitution, in the spirit of its existence, there can be no refusal by the regular profession to accept as proper subject for its consideration any proposition honestly submitted for the cure or amelioration of disease, and as proper methods for its employment any modes of dealing with disease which may have met with the approval of experience.

The failure of a system of medicine to be vindicated in its entirety by no means predicates the propriety of denying the individual elements of its practice; and while the futility of the law of similars, upon which homeopathy is founded, has been more than once demonstrated, there are individual instances of drug action apparently in harmony with its teachings, which cannot be gainsaid. The realization of a double action of a large number of medicaments, a stimulative and a depressive influence, depending upon the dosage, was made possible the earlier for the existence of homeopathy. The shield would have been held forever as a shield of gold alone, had not the black knight who looked upon its golden side been confronted by his white opponent who had only gazed upon the shimmering silver turned toward him. The good should always be acceptable; nor can one conscientiously turn from it whether its discovery be the logical outgrowth of scientific reasoning, the blind finding of empiricism, or the fortuitous conclusion from faulty premises.

The differences which are the real cause of separation of the so-called homeopathic branch from the regular medical profession lie not so much in the matter of medical thought and practice as in the assumption of this sect of a distinctive name. There are but a fractional proportion of homeopathists who are "pure" homeopaths. The majority of these practitioners repudiate daily the law of similars, and Hahnemann's *Organon* has ceased to be a text, or even a work for common consultation by them. Many openly combine their medicaments, and the dosage of the advanced members of the homeopathic branch is scarcely distinguishable from that of a large proportion of the regular profession. The higher potencies are gradually but steadily being discarded for the lower; and the triturates which insure thorough diffusion of the remedy, and consequent greater chance for absorption and action, are widely adopted by regular physicians.

In the specialties, one must scrutinize with care to distinguish the homeopath from his regular brother, unless perchance "*he trades upon a name.*" There lies the nut, uncovered from its shell. Consciously or unconsciously, with purpose or thoughtlessly, a large proportion of the homeopathic school—probably much larger than that which must be excluded from this assertion—is trading on a name, and perpetuating a schism which has no right to exist in a profession claiming to exercise all scientifically or empirically proven methods of combatting disease. No physician has a right to name himself a "regular medical practitioner" who is unwilling, where circumstances demand it for the best, to make use of medical methods whether they be drawn from the experience of a graduate, a savage, a "Christian Scientist," a homeopath, or from the investigations of the laboratory of the bacteriologist; and no physician whose honest aim is the relief of ills, and who hesitates at nothing consistent with his morality and his love for his fellowman, can be excluded from its limits. These propositions true,—and their truth lies easily within the pale of proof,—the honor of announcing that one prescribes according to any driven set of rules to the exclusion of all else, becomes the dishonor of the schismatic, and of the trader upon a name.

There is even another side to the question. Granting that, for the sake of the argument, "it is honorable to prescribe according to the law of similars," the conclusion of the editor of the *Medical Century* by no means follows that "it is equally honorable to announce that one so prescribes." The mere fact that a man has especial faith in this fad of practice, or in some obscure mode of therapeutics, by no means excludes him from the bounds of the regular profession of physicians. provided he does not systematically conceal his methods or make profit by announcement of his exclusivism. The fault lies not in the modes or the medicines employed, but in the failure to appreciate the teachings of the golden rule and the consequent neglect of its practice—not so much in the treatment of the patient, perhaps, as in the treatment of fellow physicians. It is the attempt, consciously or unwittingly, to attract attention by the exhibition of a title of exclusivism; in other words, to trade upon a name. It

It may be perfectly honorable to be a public hangman, yet it can scarcely be thought honorable to noise the fact abroad. It is honorable to labor at the anvil or at the plow, yet it is not necessarily an honor to blatantly call attention to the fact. It is honorable, when done conscientiously, to prescribe according to the law of similars; but it is actually dishonorable to noise the fact abroad and emblazon it from doors and windows to attract the sheckels of dives and pauper, and an hundred times disgraceful to him who trades upon the name without even pretending adherence to its tenets.

It is time for adjustment. The development of a spirit of reorganization, the recognition of the absence of sufficient reason for the separation of the broader minded physicians, homeopathic and regular; the recoil from a life acted under a false standard on the one side, and the fulfillment of the all-embracing destiny of the other, impress the necessity for the obliteration of the lines of dissension. And the first of all is the denial of all "pathies" by the earnest seeker for unity in his profession; there can be but one middle ground between the so-called homeopath and the misnamed allopath—that is, the regular physician. To such an one denial of association and consultation were a crime against the spirit and the letter of that code which is the highest law—"do unto others as you would have others do unto you."

